# Recent Advances in the Clinical Translation of Small-Cell Lung Cancer Therapeutics

**DOI:** 10.3390/cancers17020255

**Published:** 2025-01-14

**Authors:** Subhadeep Das, Shayak Samaddar

**Affiliations:** 1 Department of Biochemistry, Purdue University, BCHM A343, 175 S. University Street, West Lafayette, IN 47907, USA; 2 Purdue University Institute for Cancer Research, Purdue University, Hansen Life Sciences Research Building, Room 141, 201 S. University Street, West Lafayette, IN 47907, USA; 3 Eli Lilly and Company, Indianapolis, IN 46221, USA; shayak.samaddar@gmail.com

**Keywords:** small-cell lung cancer, molecular subtypes, chemotherapy, resistance

## Abstract

Small-cell lung cancer (SCLC) is a recalcitrant form of cancer, representing 15% of lung cancer cases globally. SCLC is classified within the range of neuroendocrine pulmonary neoplasms, exhibiting shared morphologic, ultrastructural, immunohistochemical, and molecular genomic features. It is marked by rapid proliferation, a propensity for early metastasis, and an overall poor prognosis. The current conventional therapies involve platinum–etoposide-based chemotherapy in combination with immunotherapy. Nonetheless, the rapid emergence of therapeutic resistance continues to pose substantial difficulties. The genomic profiling of SCLC uncovers significant chromosomal rearrangements along with a considerable mutation burden, typically involving the functional inactivation of the tumor suppressor genes TP53 and RB1. Identifying biomarkers and evaluating new treatments is crucial for enhancing outcomes in patients with SCLC. Targeted therapies such as topoisomerase inhibitors, DLL3 inhibitors, HDAC inhibitors, PARP inhibitors, Chk1 inhibitors, etc., have introduced new therapeutic options for future applications. In this current review, we will attempt to outline the key molecular pathways that play a role in the development and progression of SCLC, together with a comprehensive overview of the most recent advancements in the development of novel targeted treatment strategies, as well as some ongoing clinical trials against SCLC, with the goal of improving patient outcomes.

## Simple Summary

Small-cell lung cancer (SCLC) is an aggressive type of lung cancer, often linked to a poor prognosis for the majority of patients. Platinum–etoposide-based chemotherapy, combined with immunotherapy, constitutes the standard treatment for patients with small-cell lung cancer (SCLC). Nonetheless, SCLC often recurs and develops resistance to treatment. The development of targeted treatment options for patients with SCLC has presented challenges; however, several emerging therapies demonstrate potential efficacy. Recent progress in SCLC research has uncovered essential insights into the biological traits of the disease, which could facilitate the discovery of biomarkers. Additionally, evaluating novel therapies will be crucial for enhancing treatment outcomes for patients with SCLC.

## 1. Introduction

Lung cancer represents the leading cause of cancer-related mortality globally and exhibits the highest morbidity rates among all cancer types [1]. Small-cell lung cancer (SCLC), which accounts for around 13–15% of all lung cancer cases, is the most aggressive histologic subtype of the disease [2]. Each year, around 250,000 patients are diagnosed with small-cell lung cancer (SCLC) worldwide, with approximately 200,000 deaths resulting from the disease [1,3,4]. SCLC displays significant neuroendocrine differentiation [5,6] and primarily occurs in individuals who are current or past smokers [7,8,9]. The recalcitrant features of small-cell lung cancer (SCLC) are evidenced by aggressive disease progression, extensive metastasis, genomic instability, and unfavorable prognosis [10,11,12]. The significant molecular alterations linked to SCLC include the loss of TP53 and RB1, along with MYC amplification [8,11]. Recent comprehensive genomic analyses of small-cell lung cancer (SCLC) have revealed a substantial mutational burden along with significant chromosomal rearrangements, typically involving the loss of TP53 and RB1 in addition to MYC oncogene amplification [9,11,13,14,15]. Small-cell lung cancer (SCLC) exhibits significant sensitivity towards initial chemotherapy and radiotherapy [3,11,16]. The platinum–etoposide-based chemotherapy, in combination with immunotherapy, has served as the standard treatment procedure for patients with extensive-stage small-cell lung cancer (ES-SCLC) for several decades. Despite this, the median survival rate seldom exceeds 1 year [1,4]. The overall five-year survival rate for SCLC patients continues to be under 7% [10,17].

Recent investigations have examined the mechanisms through which small-cell lung cancer (SCLC) endures initial chemotherapy and acquires resistance [18]. In this current review, we have examined recent insights into chemoresistance in SCLC and their possible clinical implications. This review offers an in-depth investigation of SCLC, addressing its cellular origins, neuroendocrine characteristics, genetic alterations and the various subtypes based on transcriptional regulators. We also explore the mechanisms that contribute to chemotherapy resistance in SCLC. Finally, we discuss some recent novel therapeutic strategies, as well as the numerous ongoing clinical trials assessing emerging therapies for more effective targeted therapy against SCLC to enhance patient outcomes.

## 2. Cellular Origin of SCLC

There are several different cell types found in the lung epithelium [19,20]. The lining of the lung epithelia is organized into three distinct domains along the proximal to distal axis, each with its own unique structure and function [10,21]. The bronchioalveolar duct junction (BADJ) is the site where airways and alveoli connect. It is populated by bronchioalveolar stem cells (BASCs) that differentiate into both airway and alveolar cells [10,22]. The distal alveolar area is composed of squamous alveolar type 1 (AT1) cells responsible for facilitating gaseous exchange and cuboidal alveolar type 2 (AT2) cells produce surfactant to prevent the collapse of the alveoli [10,23]. Basal cells, ciliated epithelial cells, goblet cells, and Clara cells make up the majority of the cells that line the conducting airways [19,20]. The proximal airways contain basal cells, that serve as progenitor cells for ciliated, neuroendocrine (NE), as well as club cells [10,24]. AT2 cells are considered to have greater stem cell-like characteristics as they possess the capacity to self-renew and transition into AT1 cells [19,25,26]. The emergence of NE cells throughout lung organogenesis is a crucial aspect of pulmonary evolution and development. These epithelial cells are particularly abundant in fetal and neonatal lungs, underscoring their significance. The NE cells originate from versatile epithelial progenitors that are identified by the presence of the basic helix–loop–helix (bHLH) transcription factor inhibitor of differentiation 2 (ID2) [20,23]. ID2 modulates mitochondrial function; increasing its expression helps SCLC cells receive enough energy to sustain rapid cell division and growth.

Pulmonary neuroendocrine cells (PNECs) are found either as solitary cells in the proximal airways or as clusters known as neuroepithelial bodies (NEBs) at the intralobar airways. PNECs are found in various species, including primitive amphibians and mammals, but they make up a small proportion of respiratory cell types [27,28]. Numerous studies have determined that PNECs play an important role during lung development, particularly in the initial stages of lung growth and maturation by modulating amine and peptide levels, and in the fetal and postnatal periods by acting as airway chemoreceptors [27,28]. PNECs exhibit properties that are typical for both neuronal and endocrine cells. This involves the production, buildup, and release of various neurotransmitters, including serotonin, gastrin-releasing peptide (GRP), neuron-specific enolase (NSE), and bombesin [10,29]. It is worth noting that calcitonin gene-related peptide (CGRP), neural cell adhesion molecule 1 (NCAM1), and mammalian achaete-scute complex homolog 1 (MASH1/ASCL1) have significant functions in neuronal differentiation, and are prominently expressed in PNECs [10,30]. SCLC primarily originates from PNECs, but can also develop from lung epithelial cells, including basal or club cells and AT2 cells in particular instances. Researchers have identified Tuft cells as potential progenitor cells in a specific subtype of small-cell lung cancer (SCLC) [10,31]. Tuft cells, also known as brush cells, are chemosensory cells that reside in the epithelial lining of the lungs [10,32].

The development of small-cell lung cancer (SCLC) is clearly associated with loss-of-function mutations in genes such as tumor protein 53 (TP53) and retinoblastoma 1 (RB1) in PNECs, tuft, club, or AT2 cells. In addition, it is worth noting that SCLC has the ability to transdifferentiate from lung adenocarcinoma (LUAD) when certain driver mutations, like epidermal growth factor receptor (EGFR), are lost [10].

## 3. Neuroendocrine Characteristics of SCLC

High-NE tumors exhibited irregular floating clusters with a few adherent subpopulations in vitro. Alternatively, tumors with low levels of NE differentiation exhibited semi-attached, loosely aggregated, or solitary floating cell clusters [10,33]. SCLC was initially classified as a lymphosarcoma in 1879 by Harting and Hesse [34,35]; however, it was reclassified as tiny ‘oat cell’ carcinomas of the lung in 1926 [35,36]. Since then, it has been widely recognized as oat cell carcinoma [35,37,38]. SCLC is classified as a neuroendocrine pulmonary neoplasm, which is a group of tumors that have similar characteristics in terms of morphology, ultrastructure, immunohistochemistry, and molecular genomics [5,35].

The four main types of neuroendocrine pulmonary neoplasms include carcinoids (typical and atypical) and neuroendocrine carcinomas (SCLC and large-cell neuroendocrine carcinomas; LCNECs). Both neuroendocrine carcinomas are categorized as high-grade neoplasms, in contrast to normal and atypical carcinoid neoplasms, which are low-grade and intermediate grade, respectively [5,35]. All the major subtypes of lung cancer, including SCLC, adenocarcinoma, and squamous cell carcinoma, are thought to have originated from multipotent progenitor cells in the endodermal stage.

The NE expression profiles within tumors exhibit significant heterogeneity, with variations in morphological characteristics, growth features, genetic variations, along with inflammatory and immune responses [10,39]. The presence of immune cells in NE-low SCLCs is higher. In contrast, NE-high SCLCs have a minimal immune cell response [10,40]. SCLC exhibits varying levels of neuroendocrine differentiation markers, including neural cell adhesion molecule (NCAM/CD56), chromogranin, synaptophysin, and insulinoma-associated protein 1 (INSM1) [35,36,37,41]. It should be noted that a minority of small-cell lung cancers (SCLCs) do not express any standard neuroendocrine markers. Pulmonary biomarker, Napsin A, can be observed in approximately 15% of LCNEC, but it is not present in SCLC [41,42]. Thyroid transcription factor-1 (TTF-1) is a transcription factor that is found in pulmonary adenocarcinomas, thyroid tumors, and small cell carcinomas. It contains a homeodomain and is selectively expressed in these specific types of cancers. TTF-1 is expressed in approximately 90% of small-cell lung cancer (SCLC), aiding in the distinction between SCLC and non-lung neuroendocrine cancers [5,35,41,43]. Elevated Ki67 (MIB-1) labeling index represents a characteristic feature of small-cell lung cancer (SCLC) and is useful in differentiating it from neuroendocrine cancers of low and intermediate grades [41,43]. Additional markers utilized for characterizing SCLC include B-cell lymphoma 2 (BCL2; frequently expressed), P16 nuclear staining, tyrosine-protein kinase KIT (CD117/c-KIT), retinoblastoma protein (P-RB; consistently negative), and homeobox protein orthopedia (OTP; consistently negative) [41,43].

## 4. Subtypes of SCLC

The initial indication of distinct SCLC subtypes emerged from an examination of morphological variations across SCLC cell lines. SCLC cells that belong to the ‘classic’ subtype primarily develop as spherical clusters of floating cells, which may or may not exhibit central necrosis. In contrast, cells coming from the ‘variant’ subtype tend to grow in the form of loosely adherent aggregates or in the form of a more tightly adherent monolayer [9,44].

**Figure 1:**
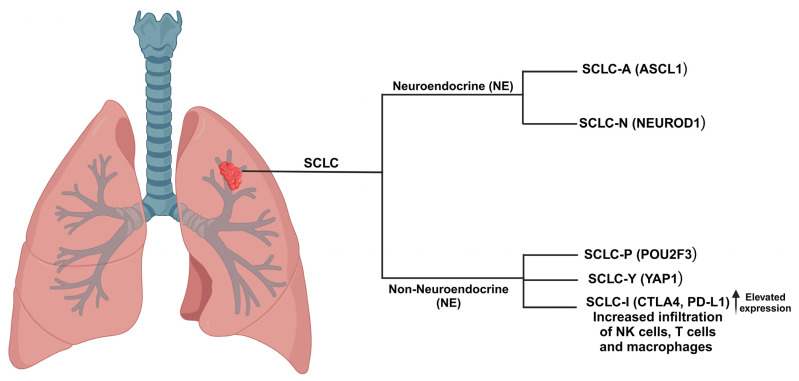
Different subtypes of small-cell lung cancer. SCLC can be categorized into specific subtypes defined by the varying expression of four essential transcriptional regulators: achaete-scute homolog 1 (ASCL1; also referred to as ASH1) (SCLC-A subtype), neurogenic differentiation factor 1 (NEUROD1) (SCLC-N subtype), yes-associated protein 1 (YAP1) (SCLC-Y subtype), and POU class 2 homeobox 3 (POU2F3) (SCLC-P subtype). The most recent nomenclature designates a subtype known as ‘inflamed’ small-cell lung cancer (SCLC-I).

Current genome-wide association investigations in small-cell lung cancer (SCLC), involving comprehensive molecular analyses of cancer cell lines, genetically engineered mouse models (GEMMs), patient-derived xenografts (PDXs), and primary human tumors, indicate a model of distinct subtypes characterized by the differential expression of four key transcriptional regulators: achaete-scute homolog 1 (ASCL1; also known as ASH1) (SCLC-A subtype), neurogenic differentiation factor 1 (NEUROD1) (SCLC-N subtype), yes-associated protein 1 (YAP1) (SCLC-Y subtype), and POU class 2 homeobox 3 (POU2F3) (SCLC-P subtype) [17,45,46] (Figure 1).

The latest nomenclature identifies a subtype termed ‘inflamed’ small-cell lung cancer (SCLC-I) and another subtype characterized by the elevated expression of the neuroendocrine transcription factor ATOH1 [3,10,45,47,48] (Figure 1). Additional investigations into this finding are essential. The relationship among these subtype-specific transcription factors encompasses varying levels of neuroendocrine differentiation. This may reveal subtype-specific therapeutic vulnerabilities of clinical significance [10,17]. In a comprehensive multiomic evaluation of 437 SCLCs, primarily metastatic, the overall distribution of samples was as follows: 35.7% coming from the A subgroup, 17.6% coming from the N subgroup, 6.4% coming from the P subgroup, 21.1% coming from the Y subgroup, and 19.2% coming from mixed subgroups [9,49]. The A and N subtypes are typically associated with elevated expression of neuroendocrine markers, while the P and Y subtypes exhibit reduced levels of these markers [9].

### 4.1. Neuroendocrine Subtypes

ASCL1 is a basic helix–loop–helix (bHLH) transcription factor that is typically expressed in fetal pulmonary neuroendocrine cells (PNECs), essential for their differentiation [19,50]. ASCL1 plays a role in the regulation of chromatin architecture. Its association with cis-acting enhancer elements promotes the formation of open chromatin, enhances chromatin accessibility thereby facilitates gene expression and differentiation. Reports indicate that the upregulation of ASCL1 additionally fosters neuronal development and regeneration in young mice [19,51]. Expression of ASCL1 is essential for the survival of SCLC tumors that express it [52]. The expression of ASCL1 is correlated with classical SCLC morphology [33,44,53]. Knockdown of ASCL1 leads to G2-M-phase cell cycle arrest, impeding both anchorage-dependent and independent growth, thus preventing tumor development in vitro [19,50,52]. ASCL1 modulates the level of expression of downstream oncogenic targets, which include L-MYC, SRY-box transcription factor 2 (SOX2), B-cell lymphoma 2 (BCL2), RET, and NFIB, along with the lung development genes FOXA2 and TTF-1. The homeobox protein Nkx2.1 (NKX2-1/TTF1) is a transcription factor found in club and alveolar type 2 (AT2) cells. TTF1 plays a critical role in NE cell development and exhibits increased expression in SCLC-A compared to SCLC-N [54].

ASCL-1 also regulates the expression of NE markers such as NCAM1, insulinoma-synaptophysin (SYP), insulinoma associated protein 1 (INSM1), chromogranin A (CHGA), and CGRP [10,55]. Furthermore, ASCL1 influences components of the Notch pathway, such as delta like canonical Notch ligand 3 (DLL3) [10,55,56]. Studies revealed that small-cell lung cancers (SCLCs) with high levels of ASCL1 may be further classified into two categories based on the expression of hairy and enhancer of split-1 (HES1): SCLC-A (HES1-low) and SCLC-A2 (HES-1 high), respectively. As a downstream target of the Notch signaling pathway, HES1 plays a crucial role in regulating cellular proliferation, differentiation, invasion, cancer stem cell (CSC)-like characteristics, and the development of tumors. Analysis of clinical data indicated that overexpression of ASCL1 serves as a negative prognostic factor in early-stage SCLC patients and correlates with unfavorable outcomes in surgically resected SCLCs [10,57]. A separate study examining the development of resistance in EGFR-mutant non-small-cell lung cancers (NSCLCs) to EGFR inhibitors revealed that resistance emerged through the transformation of NSCLC to small-cell lung cancer (SCLC). This transformation was associated with cells that possess RB1 and TP53 mutations and exhibit elevated levels of ASCL1 [19,58].

NEUROD1 is a bHLH transcription factor that plays a role in the differentiation of neuronal, neuroendocrine, and pancreatic beta cells [55,59]. Unlike ASCL1-based SCLC tumors, NEUROD1-high tumors exhibit reduced NE expression and display variant morphology expressing lower levels of ENO2 while lacking GRP and DDC expression [10,13,44,53,55]. It has been demonstrated that NEUROD1 expression is essential for the survival, proliferation, and migration of NEUROD1-positive small-cell lung cancers [19,60,61]. Several mechanistic investigations have shown that NEUROD1 effectors boost the survival, migration, and proliferation of small-cell lung cancer (SCLC) cells by activating cell surface receptor tyrosine kinase tropomyosin-related kinase B (TRKB) and neural cell adhesion factor (NCAM1). Both TRKB and NCAM1 promote invasive behavior in NE lung tumors, which facilitates metastasis [10,62,63]. By modulating the interaction between fibroblast growth factor (FGF) and receptor (FGFR), NCAM1 expression promotes cell–matrix and neurite outgrowth. It is worth noting that amplification of FGFR1 occurs in 5.6% of small-cell lung cancers (SCLCs), indicating a potential therapeutic target [2,10,64]. Conversely, the overexpression of TRKB leads to changes in the expression of molecular factors involved in epithelial-to-mesenchymal transition (EMT), which includes the downregulation of CDH1 as well as the upregulation of Twist [10,65]. Conversely, the overexpression of TRKB leads to changes in the expression of molecular factors involved in epithelial-to-mesenchymal (EMT) transition [10,65]. Furthermore, the transcriptional targets of NEUROD1 encompass C-MYC, as opposed to L-MYC, which is a target of ASCL1 [55], along with the receptor tyrosine kinase insulin-growth factor receptor 1 (IGF1R) [55,56].

### 4.2. Non-Neuroendocrine Subtypes

POU2F3, a class II POU domain transcription factor, is recognized as a predominant regulator of both normal and malignant chemosensory cells called tuft cells [31,66,67,68,69]. These cells are also referred to as brush cells in the lung epithelium [66,67]. Tuft cells constitute a unique lineage separate from the neuroendocrine cell lineage. Analogous to neuroendocrine cells, they react to external stimuli by secreting active substances that govern the surrounding local microenvironment [19,31,70,71]. POU2F3 expression is observed in a subset of small-cell lung cancers (SCLC-P) that display a non-neuroendocrine phenotype. However, these tumors may also express reduced levels of neuroendocrine markers, including gastrin-releasing peptide (GRP) and calcitonin-related polypeptide alpha (CALCA), in addition to genes typically associated with tuft cells, such as the lineage-specific transcription factors SOX9 and ASCL2 [19,31,45,46]. Additional significant markers in SCLC-P comprise IGF-1R and growth factor independence 1B (GFI1B) [45,62]. Reports suggest SCLC-P demonstrates enhanced survival outcomes in comparison to other subtypes [13,31,40].

YAP1 is a crucial transcriptional regulator of the Hippo growth signaling pathway. It is essential in modulating organ size through cell-to-cell contact-dependent mechanisms that govern cell proliferation and apoptosis [72]. It also serves as an oncogene, with its overexpression triggering EMT [73,74]. The expression of YAP1 is observed in a specific subset of SCLCs that lack GRP and NCAM expression displaying a non-neuroendocrine phenotype [75]. SCLC cells that express YAP1 (SCLC-Y) are likely to exhibit an increased adherent phenotype and also show elevated levels of laminin and integrin expression [75,76]. Reports indicate that YAP1-driven repression of the ajuba LIM protein (AJUBA) correlates significantly with shorter overall survival (OS) in patients with SCLC [10,77]. The notion of a YAP1-high subtype is currently under scrutiny, and its clinical setting is still ambiguous. YAP1 expression is observed at moderate levels across all subtypes [10,46,78,79]. It remains unclear whether YAP1 functions primarily as a transcriptional regulator of this phenotype or merely as a marker for it [80].

Nonetheless, a specific group of SCLCs characterized by elevated YAP1 expression and lacking POU2F3 expression, referred to as the inflamed SCLC subtype (SCLC-I), displays both mesenchymal and inflammatory phenotypes [79]. This subtype is marked by elevated levels of expression of programmed death ligand 1 (PD-L1), programmed cell death protein 1 (PD-1), human leukocyte antigens (HLAs), and cytotoxic T-lymphocyte-associated protein 4 (CTLA4). The subtype is marked by the infiltration of immune cells, including an enhanced level of macrophages, NK cells, and T-cells [79]. SCLC-I shows reduced expression of the epithelial marker CDH1 while exhibiting elevated expression levels of the mesenchymal markers such as vimentin and AXL [79]. It has been proposed to substitute the YAP1 subtype with the inflamed SCLC subtype (SCLC-I) [10,79].

## 5. Genetic Alterations in SCLC

The genetic alterations found in cancers play a crucial role in the development of tumors [28]. In order to find novel therapeutic targets and provide more effective medications, recent studies have concentrated on comprehending the genetic foundation of SCLC [81]. The present state of comprehensive whole-genome studies on oncogenic driver mutations for SCLC is hindered by the scarcity of patient samples compared to other types of cancer [28]. Genome-wide investigations have confirmed that tobacco smoking is a significant factor in the development of nearly all types of lung cancer [10,82,83].

SCLC is highly correlated with smoking, with approximately 97.5% of these tumors occurring in individuals who currently smoke or have smoked in the past [9,84,85,86]. Additionally, to the rare occurrence of de novo small-cell lung cancers (SCLCs) in non-smokers (<2%), some SCLCs are believed to develop through the transformation of lung adenocarcinomas (LUADs), a type of non-small-cell lung cancer (NSCLC) that carries EGFR mutations or ALK alterations (~14%) [9,87,88,89]. The study found a correlation between current or former heavy smoking and higher levels of tumor mutational burden (TMB), heterogeneity, and driver mutations [10,90]. SCLC is associated with a TMB of approximately 9.9 mutations per megabase. The most prevalent form of base substitution in SCLC is C:G > A:T transversions, which are caused by DNA damage mechanisms that arise from smoking [9,91].

A comprehensive genome-wide analysis was conducted on 110 surgically removed SCLC tumors [9,13]. A biallelic inactivation of the tumor-suppressor genes TP53 and RB1 was seen in the majority of SCLCs (~95%) in the investigation [13,28], and TP73 mutation was also identified [13]. The presence of somatic genomic rearrangements in exons 2 and 3 of TP73 was noted. These rearrangements give rise to oncogenic transcription factors that exert a dominant-negative impact on wild-type p53 family members [13,28,92]. The notion was later confirmed in a genetically engineered mouse model (GEMM), where the deletion of these two genes is associated with the formation of tumors that resemble small-cell lung cancers (SCLCs) observed in patients [9,34]. The majority of cases confirm the loss of TP53 in 75–90% of patients [10,93] and RB1 in nearly 100% [10,94].

The molecular signatures observed in SCLC are distinct compared to those found in NSCLC, where a range of oncogenic driver mutations/fusions are prevalent, such as EGFR, KRAS, ALK, BRAF, RET, ROS1, MET, NTRK1-3, and HER2/ERBB2 [3,35,95]. Nevertheless, the loss of RB1 and/or TP53 is not universally observed in SCLC. According to reports, approximately 5% of small-cell lung cancers (SCLCs) have normal RB1 and/or TP53 genes [9,96]. The p53 protein plays a crucial role in maintaining genomic stability, regulating apoptosis, and inhibiting angiogenesis [28,97]. The initiation event in SCLC development is attributed to gene alteration in P53 [28,98]. RB1 is commonly inactivated in a vast majority of SCLC cases [13,28]. It was recently discovered that RB1 might inhibit the pluripotency processes in somatic cells of SCLC patients through direct interactions with transcription factors including Nanog, Oct4, and Sox2 [28,99]. The loss of RB1 can activate transcription factors while enhancing pluripotency characteristics, increasing the aggressiveness of SCLC cells in reprogramming and tumorigenesis [28,99]. In addition, a strong correlation was observed between the loss of RB1 in SCLC and the activation of EZH2 [100]. There exists a significant correlation between the high expression of EZH2 in lung cancer and tumor growth [28,101].

Various other genes frequently altered in small-cell lung cancer (SCLC) include RBL1 and RBL2 from the retinoblastoma family, NF1B and PTEN tumor suppressors, NOTCH1, NOTCH2, NOTCH3, and NOTCH4 from the Notch signaling pathway, as well as ARID1, CREBBP, EP300, KMT2D, and SMARC4 that involved in regulating chromatin integrity. These genetic alterations contribute to the aggressive nature of SCLC [9,35,102]. The transcription factor, NFIB, plays a crucial role in the development of the lung, kidney, and brain during embryonic stages [10,57]. The amplification of NFIB is frequently observed in SCLC promoting the development, advancement, and metastasis of SCLC. Elevated NFIB levels are associated with the expansion of a poorly differentiated subpopulation of invasive tumors that lack E-cadherin (CDH1) expression. This correlation is linked to poorer patient survival [10,103]. The aberrant expression of critical genes that govern cell proliferation and survival may be caused by recurrent mutations in chromatin remodeling genes, including ARID1A, ARID1B, and SMARCA4. This could give rise to the development of SCLC [35]. Another study found that SCLC may be caused by recurrent mutations in STK11 (1.7% of cases), KEAP1 (∼3% of cases), and genes associated with the PI3K pathway (PTEN (9.9% of cases), PIK3CA (5.6%), and RICTOR (5.6%)). Interestingly, the researchers found that a small proportion of SCLCs tested positive for HPV. In contrast to the 1.8% of cancers with TP53 and RB1 mutations, 12.7% of tumors having wild-type TP53 and/or RB1 are HPV+. The relationship between the events is still under investigation [9,102].

The amplification of MYC proto-oncogenes, specifically C-MYC, L-MYC, and N-MYC, is observed in approximately 20% of patient samples [28,104]. The proteins belong to the superfamily of bHLH leucine zipper transcription factors that specifically bind to the canonical E-box DNA element [10,105]. The MYC family consists of a group of regulatory genes that play a critical role in various cellular processes, including cell cycle progression, apoptosis, and cell transformation. The amplification of MYC can result in elevated levels of the MYC protein, which subsequently activates genes related to cell cycle progression, leading to elevated cell proliferation and metabolism. The transcription of AURKA is induced by MYC, resulting in the production of Aurora A kinase. This kinase plays a crucial role in regulating mitosis and SCLC tumor progression [9,106]. In a GEMM of SCLC, the researchers found that overexpression of MYCN was linked to resistance of SCLC against platinum-based therapies. In addition, a comprehensive CRISPR–Cas9 screening revealed that the deubiquitinase USP7 is a synthetic vulnerability associated with MYCN. The inhibition of USP7 through pharmacological inhibitors restored the sensitivity of small-cell lung cancers (SCLCs) to chemotherapy [9,107].

The occurrence of chromosomal translocations, insertions, and deletions often leads to the formation of gene fusions, which play a significant role in the development of solid tumors [108]. The extent of their role in SCLC is still being determined. SCLC-derived cell lines and tumors commonly exhibit a recurrent RLF-MYCL fusion [9,109,110]. A mouse model of SCLC driven by Rlf–Mycl was established, demonstrating how this fusion enhances the transformation, advancement, and dissemination of SCLC cells to various organs [9,111].

## 6. Mechanisms of Resistance

Drug resistance in SCLC is a multifaceted and intricate phenomenon, marked by alterations in both tumor cells and the tumor microenvironment (TME) via several pathways that lead to resistance against chemotherapy [1]. In recent years, numerous studies have contributed to the understanding of the molecular mechanisms underlying acquired resistce in SCLC [10].

### 6.1. DNA Damage Response Mechanisms

DNA damage, which can be induced by external damaging agents, necessitates that tumor cells preserve genomic integrity in order to ensure continued cell proliferation and survival [10,112]. Poly (ADP-ribose) polymerase (PARP) enzymes are activated through the binding of single-strand DNA breaks (SSBs). They are part of a family of DNA damage repair (DDR) proteins that play a crucial role in the recognition and repair of DNA breaks, in addition to engaging in transcriptional regulation and chromatin remodeling [1,112]. PARP facilitates poly-ADP ribosylation (PARylation) of various substrates, subsequently recruiting proteins that promote DNA repair mechanisms. Subsequently, poly(ADP)-ribose glycohydrolase (PARG) and additional enzymes metabolize PAR groups, which is essential for efficient DNA repair [112]. Without SSB repair by PARP1, the replication fork halts, leading to double strand breaks that necessitate repair through homologous recombination (HR) or non-homologous end joining (NHEJ). Improper repair of double-strand breaks (DSBs) can lead to replication errors, including mutations, deletions, chromosomal translocations, and amplifications, which eventually result in cell death, senescence, or malignant transformation [113]. Byers’s lab initially identified PARP as a promising therapeutic target in small-cell lung cancer [114]. The expression of PARP1 mRNA and protein increased significantly in SCLC cell lines as in comparison to NSCLC. Furthermore, immunohistochemistry (IHC) study of tissue microarrays confirmed the elevated expression of PARP1 protein [114]. Numerous preclinical studies have indicated that the inhibition of DDR proteins such as PARP increases the antitumor immune response facilitated by PD-L1 inhibition via T cell-mediated effects [10,115,116]. The primary mechanisms through which PARP inhibitors exert their effects involve locking the enzyme at single-strand breaks (SSBs) and hindering PARylation and binding of PARP to DNA [1,117].

A study identified a strong correlation between high-mobility group box protein B1 (HMGB1) and chemoresistance in small-cell lung cancer (SCLC). HMGB1 initiates PARP1 self-modification, enhancing its association with microtubule-associated protein/light-chain 3 (LC3), leading to nucleophagy. This leads to chemoresistance in SCLC [10,118].

The loss of RB1 in SCLC results in a disruption of E2F1 inhibition, which subsequently causes a rise in the levels of several key mediators of DDR, including PARP1 and notably the CHK1 protein. CHK1 is a crucial serine threonine kinase that serves as the principal mediator of DNA damage induced cell cycle [1,119,120,121,122]. A biomarker examination demonstrated significantly elevated CHK1 protein expression in small-cell lung cancer (SCLC) relative to normal lung tissue [123]. CHK1 inhibitors have the potential to enhance DNA damage effects, and preliminary preclinical data suggest that CHK1 represents a promising target for small-cell lung cancer (SCLC), particularly in tumors exhibiting cMYC protein overexpression [123]. Prexasertib (LY2606368), a second-generation CHK1 inhibitor, has been studied both as a monotherapy and in combination with chemotherapy in models of platinum-sensitive and -resistant small-cell lung cancer (SCLC) [124].

The Aurora A protein is a member of the Aurora protein family and is crucial in several mitotic processes including chromosomal alignment, segregation, centrosome maturation, and cytokinesis [1,125]. Prior research indicates that small-cell lung cancers (SCLCs) exhibit significant sensitivity to Aurora A kinase inhibition, resulting in multiple clinical trials evaluating this treatment in SCLC [126,127,128,129,130,131,132]. A new study demonstrated that the Aurora A inhibitor can effectively inhibit SCLC cell proliferation and induce G2/M-phase arrest.

In SCLC, the significant genomic instability, frequent amplification and overexpression of oncogenes, together with the elevated expression of lineage specific transcription factors, trigger replication stress (RS) [133,134]. Replication stress (RS) is a prevalent feature linked to activated oncogenes or inactivated tumor suppressor genes, which accelerates the pace of S-phase entry followed by subsequent disruption of the orderly DNA replication process in cancers [135]. Ataxia telangiectasia-mutated and rad3-related (ATR) serves as the major responder to replication stress (RS). The inhibition of ATR can trigger cell death while enhancing the efficacy of chemotherapy, specifically with topoisomerase I (TOP1) [136]. The combination of the first-in-class, ATP-competitive inhibitor of ATR (M6620) with topotecan was investigated in a phase I study including SCLC patients who experienced a relapse following at least one previous course of systemic therapy [1,137].

### 6.2. Cancer Stem Cells and Their Associated Signaling Cascades

**Figure 2:**
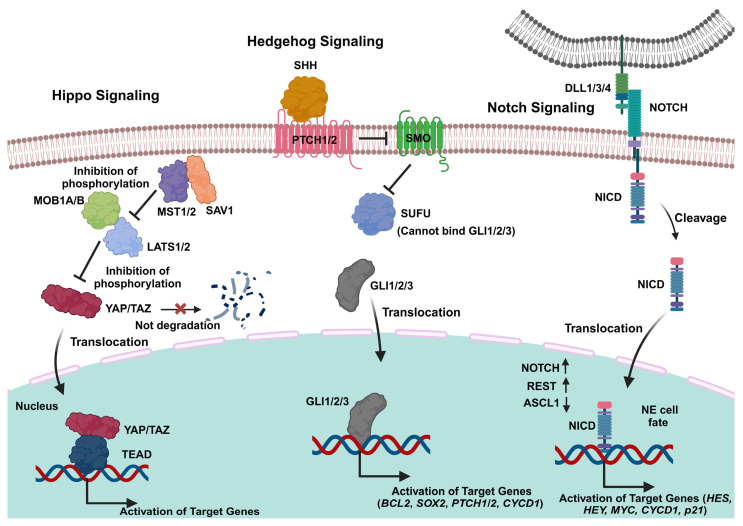
Different signaling pathways associated with the progression of SCLC. The Hippo, Hedgehog, and Notch signaling pathways become significantly altered, leading to the activation of target genes, which thus leads to SCLC.

Cancer stem cells (CSCs), known as tumor-initiating cells, represent a tiny subset of malignant cells that possess unlimited proliferative capacity. They demonstrate a significant ability for self-renewal, metastatic spread, and resistance to therapy interventions [138,139]. Cancer stem cells (CSCs) exhibit numerous traits similar to those of embryonic stem cells [1,140,141]. CSCs arise from normal cells by acquiring stem cell-like traits, primarily as a result of EMT, which plays a key role in the development of fibrosis alongside various malignant transformations [10,142,143,144,145]. Stem cell signaling enhances intratumoral heterogeneity and fosters EMT traits in SCLC [146]. They have the capacity to persistently stimulate one or more signaling pathways implicated in the process, such as the Hedgehog, Hippo, and Notch pathways, which may be heightened in small-cell lung cancer (Figure 2) [1,140,141].

#### 6.2.1. Notch Signaling

Notch signaling modulates stemness, exhibiting either pro-tumorigenic or tumor suppressor functions in small-cell lung cancer (SCLC) [10]. Research indicated that SCLC cells exhibited a notable growth arrest associated with active Notch1/2, which was linked to the activation of p21 in addition to the arrest of the G1 cell cycle [1,13]. Knockdown of Notch could result in enhanced cellular proliferation, which contributes to the antitumor activity observed in SCLC cells [147]. Notch interferes with NE differentiation and is suppressed in most SCLCs [148]. Activation of the Notch pathway occurs through the binding of DLL1/3/4 or Jagged-like ligands (JLL1/2) to their specific receptors (Notch 1–4), resulting in a conformational change. Furthermore, the cleavage of the Notch receptor is enhanced when γ-secretase facilitates the release of the Notch intracellular domain (NICD) from the cell membrane [149,150]. This is followed by the translocation of NICD into the nucleus, where it engages with the DNA thereby promoting the transcription of the regulatory proteins HES1 and hairy and enhancer of split-related protein 1 (HEY1) [151]. The transcriptional repressors HES1 and HEY1 function by inhibiting ASCL1, which in turn activates the production of Notch ligands [152]. The endogenous induction of Notch signaling, along with the loss of neuroendocrine differentiation, leads to a transition from a neuroendocrine to a non-neuroendocrine fate, partially facilitated by the RE1 silencing transcription (REST) co-repressor [10,153]. DLL3, an inhibitory ligand of the Notch pathway, is expressed in accordance with ASCL1, functions as a dominant-negative inhibitor of Notch signaling [154]. It is significantly elevated and aberrantly expressed in small-cell lung cancer (SCLC) and other high-grade neuroendocrine tumors [1,155]. The expression of DLL3 downregulates and inhibits Notch signaling during the development of neuroendocrine tumors [156]. DLL3 expression enhances the invasive and migratory properties of small-cell lung cancer (SCLC) in preclinical models [157]. Rovalpituzumab tesirine is an antibody–drug conjugate (ADC) that targets DLL3 and is used in conjunction with CKIs in select treatment regimens for small-cell lung cancer (SCLC) [158]. Tarextumab (OMP-59R5) is a fully human monoclonal IgG2 antibody that selectively interferes with signaling through Notch2 and Notch3. Its efficacy was initially investigated in small-cell lung cancer (SCLC) allografts and patient-derived tumor xenografts (PDXs), followed by clinical application together with chemotherapy [159,160]. Tarextumab has been demonstrated to play a significant role in delaying chemoresistance in these PDX models [159,160].

#### 6.2.2. Hedgehog Signaling

The Hedgehog (HH) signaling is crucial for governing differentiation and proliferation during the process of embryonic development. It plays a role in early lung development via epithelial–mesenchymal interaction [161,162]. The paracrine Hedgehog (HH) ligand comprises sonic hedgehog (SHH), Indian Hedgehog (IHH), and desert Hedgehog (DHH). The isoforms interact with the transmembrane receptors such as patched 1 (PTCH1) and PTCH2, thereby reducing the inhibiting impact on smoothened (SMO) [163]. The activated signaling cascade inhibits suppressor of fused (SUFU), which in turn activates the glioma-associated oncogene family (GLI1, GLI2, and GLI3). As a result, HH target genes, such as SOX2, undergo active transcription [164].

Reports indicate that the expression of the signaling proteins in the sonic Hedgehog pathway is elevated in small-cell lung cancer (SCLC), thereby playing a significant role in the development and proliferation of SCLC [165]. The selective Hedgehog pathway inhibitor, Vismodegib (GDC-0449), inhibits Hedgehog signaling through its binding to SMO, thereby preventing the activation of Hedgehog target genes [166].

#### 6.2.3. Hippo Signaling

The Hippo signaling pathway regulates the equilibrium between cell proliferation and apoptosis [10,167]. The regulation of Hippo signaling occurs through a cascade of phosphorylation events. Hippo signaling facilitates the phosphorylation of the adaptor protein Salvador homolog 1 (SAV1) through mammalian sterile 20-like kinases 1 and 2 (MST1/2). This process subsequently activates the adaptor protein MOB kinase activator 1 (MOB1A/B) along with the large tumor suppressor kinases 1 and 2 (LATS1/2) through phosphorylation [10,167]. Subsequently, YAP1 and TAZ, whereby the latter is a transcriptional coactivator that is homologous of the former, undergo phosphorylation, which leads to their destruction and then to transcriptional inhibition. In the absence of Hippo signaling, the phosphorylation cascade does not occur. This results in the translocation of the YAP1/TAZ complex into the nucleus, thereby engaging with the TEA domain transcription factor (TEAD) [168], resulting in transcriptional activation, which contributes to the expansion of CSC populations. These factors trigger the development of solid tumors, advancement of cancer, and resistance to chemotherapy, mainly in non-neoplastic small-cell lung cancer [169].

### 6.3. Epigenetic Reprogramming

**Figure 3:**
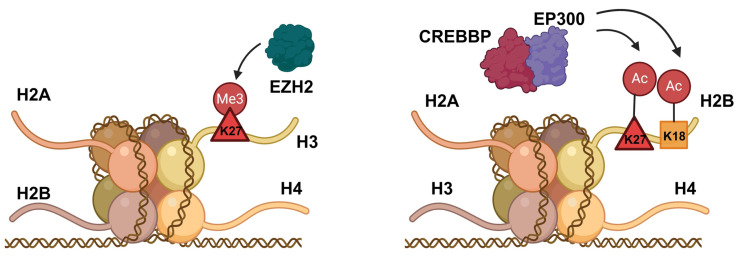
Epigenetic reprogramming leading to SCLC. Two important chromatin regulators, EZH2 and the CREBBP/EP300 complex, target the Histone 3 protein’s amino acids K27 and K18, respectively.

Epigenetic changes encompass post-transcriptional modifications that significantly influence gene expression [170]. Epigenetic variations, including DNA methylation, chromatin remodeling, and histone modification, are linked to cancer development [1,170]. EZH2, a histone methyltransferase, functions as a crucial regulator of transcription that influences DNA methylation through chromatin modification along with the induction of DNA methyltransferases (DNMTs) through targeting CpG islands [10,171] (Figure 3). EZH2 is an oncogene with elevated expression in small-cell lung cancer (SCLC) and plays a significant role in SCLC chemoresistance and immune evasion [1,172,173]. Overexpression of EZH2 results in the epigenetic silencing of transforming growth factor-β (TGF-β) receptor type 2 together with the repression of apoptosis. This leads to changes in DNA methylation that facilitate the progression of SCLC by inhibiting the TGF-β-Smad-ASCL1 pathway [174]. A separate study found that taurine-upregulated gene 1 (TUG1) regulates the activity of LIMK2b (a splice variant of LIM-kinase 2) by binding to the enhancer of EZH2, thereby mediating chemoresistance in SCLC [1,175]. In SCLC, EZH2 activation has been linked with the deletion of gene copies and the loss of functional mutations in RB1, which encodes the E2F repressor pRB [101]. The combination of EZH2 inhibition together with chemotherapy is presently under investigation in a phase I/II clinical trial involving patients with recurrent small-cell lung cancer (SCLC) [3].

The CREB binding protein gene (CREBBP) is among the most frequently mutated genes in small-cell lung cancer (SCLC) [54]. CREBBP serves as a transcriptional coactivator within the Wnt/β-catenin signaling pathway and acts as a histone acetyltransferase (HAT) [176]. The E1A binding protein p300 gene (EP300) which acts as a partner of CREBBP, is often inactivated or mutated concurrently with CREBBP. Deletion of CRB/EP300 decreases histone acetylation together with transcription of cellular adhesion genes, thereby promoting tumorigenesis [10,54,177] (Figure 3). Research also indicates that lysine demethylase 6A (KDM6A), which exhibits a strong affinity for KMT2A binding, functions as an epigenetic modifier that influences chromatin accessibility and regulates ASCL1-to-NEUROD1 subtype switching [178].

The bromodomain and extra terminal domain (BET) family proteins are crucial transcriptional regulators that interact with multiple chromatin modifiers, including HATs and HDACs [179]. They modulate MYC expression and amplification in small-cell lung cancer (SCLC) [180]. Multiple initial clinical trials are currently being conducted to investigate the preliminary efficacy and safety of BET inhibitors in individuals with SCLC [181].

Recent studies indicate that protein kinase A (PKA) serves as a predictive factor in SCLC. PKA consists of a tetrameric structure comprising two regulatory subunits and two catalytic subunits. The dissociation of regulatory subunits from cyclic AMP (cAMP) triggers the kinase (PKA-Ca), facilitating and sustaining the progression of small-cell lung cancer (SCLC). PKA-Ca is identified as the predominant catalytic subunit in small-cell lung cancer (SCLC), with 17.5% of SCLC cases exhibiting induction of the PKA/CREB (cAMP response element-binding protein) pathway. A preclinical study demonstrated that reduced PKA-Ca activity can inhibit SCLC in mouse models [45]. The PI3K/AKT/mTOR pathway is linked to the proliferation, migration, and development of SCLC [182]. Genetic modification of the PI3K/AKT/mTOR pathway have been identified as a potential therapeutic strategy for small-cell lung cancer (SCLC) [183,184]. The involvement of PI3K-dependent kinase 1 and mTORC2 in phosphorylating particular serine/threonine residues is critically important for the activation of AKT [185]. mTOR activation may also impact the expression of metabolic genes [186]. Various PI3K and mTORC1/2 inhibitors, whether used individually or in combination, were developed for SCLC patients in a phase I study [187].

Chemotherapeutic agents are generally recognized for their primary mechanism of inducing apoptosis to achieve tumoricidal effects. Consequently, the evasion of apoptosis, marked by an increase in cellular anti-apoptotic functions like the elevated levels of anti-apoptotic gene expression, could have a role in the development of drug resistance in cancer cells [11,188,189]. KCNJ2/Kir2.1 is a potassium channel that plays a crucial role in regulating cellular excitability while maintaining the resting membrane potential [190]. In SCLC, KCNJ2/Kir2.1 promotes growth and chemoresistance through interaction with multidrug resistance protein 1 (MRP1/ABCC1) and induces cell cycle arrest; this blocks drug-induced apoptotic effects [11,191]. Similarly, MRP1/ABCC1 increases substantially in chemo-resistant SCLC cells and is involved in facilitating the development of the chemoresistance phenotype [11,192,193,194]. Other ABC transporters, including ATP-binding cassette sub-family B member 1 (ABCB1), a P-glycoprotein (P-gp), have been linked to resistance against etoposide (a topoisomerase II inhibitor), while ATP-binding cassette sub-family G member 2 (ABCG2) has been associated with resistance to SN-38, the active metabolite of irinotecan (a topoisomerase I inhibitor) in SCLC cell lines. In each resistant cell line, blocking ABCB1 or ABCG2 resulted in synergistic apoptotic effects and increased drug sensitivity in resistant SCLC cells. The results of this study indicated that the ABC transporter inhibitors, elacridar and tariquidar, were effective in restoring sensitivity to etoposide or SN-38 in both in vitro and in vivo studies. Additionally, these inhibitors promoted apoptotic activity and G2-M arrest in resistant SCLC cells [195].

The Bcl-2 protein family also participates in mediating cellular apoptosis. The increased expression of Bcl-2 in small-cell lung cancer (SCLC) correlates with a negative prognosis [196]. Numerous studies indicate that inhibiting both the Bcl-2 and PI3K/mTOR pathways demonstrates synergistic effects in SCLC [197]. At present, numerous clinical studies are exploring the role of additional Bcl-2 inhibitors, such as ABT-263 [198].

## 7. Emerging Therapies for Small-Cell Lung Cancer

The optimization of therapy for SCLC continues to pose challenges; however, recent trial outcomes and approvals of drugs provide a sense of positive outlook [199]. Focusing on finding biomarkers and assessing novel treatments will be essential to enhancing treatment results for SCLC patients [199]. In the current section, we will discuss some recent clinical trials targeting SCLC (Table 1).

### 7.1. PD-L1 Inhibitors

The integration of immune checkpoint inhibitors, particularly PD-L1 inhibitors, into the treatment regimen for extensive-stage small-cell lung cancer (SCLC) has significantly altered the clinical landscape. Recent studies have demonstrated that combining these inhibitors with standard chemotherapy can enhance survival outcomes for patients with this aggressive cancer type. The KEYNOTE-604 trial, a pivotal phase III study, evaluated the efficacy of pembrolizumab in combination with etoposide and platinum-based chemotherapy. The results indicated that patients receiving pembrolizumab experienced a significant improvement in overall survival (OS) and progression-free survival (PFS) compared to those receiving placebo. Specifically, the median OS for the pembrolizumab group was reported to be 12.9 months, compared to 10.5 months for the placebo group, illustrating a clear survival advantage. The trial also noted that the addition of pembrolizumab did not introduce unexpected toxicities, with adverse events being consistent with those observed in previous studies [200].

Similarly, the IMpower133 trial assessed the combination of atezolizumab with carboplatin and etoposide. This study reported a median OS of 12.3 months for the atezolizumab group versus 10.3 months for the control group, highlighting the potential of atezolizumab to improve survival outcomes in extensive-stage SCLC. The safety profile was also favorable, with manageable adverse events, reinforcing the role of atezolizumab as a viable option in first-line therapy for SCLC [201].

The CASPIAN trial further supported these findings by evaluating durvalumab in combination with platinum–etoposide in previously untreated ES-SCLC patients. The results indicated a median OS of 13.0 months for the durvalumab group compared to 10.3 months for the chemotherapy-only group, demonstrating the efficacy of this PD-L1 inhibitor in enhancing survival rates [202]. A phase II trial (NCT04701307) assessed the effectiveness of a combination therapy involving the anti-PD-1 monoclonal antibody dostarlimab along with the PARP inhibitor niraparib in patients with extensive-stage SCLC who had previously undergone treatment [10,203].

Moreover, multiple meta-analyses of several trials evaluating combination therapies of PDL1 report that PDL1 inhibitor in combination with chemotherapy provided a significant survival benefit relative to chemotherapy alone [204,205,206]. This analysis further solidified the evidences supporting the use of PD-L1 inhibitors in combination with chemotherapy in improving clinical outcomes for patients with extensive-stage SCLC.

### 7.2. Topoisomerase I Inhibitors

Topotecan is the most extensively studied TOP1 inhibitor for SCLC. It was the first approved second-line treatment option for SCLC, especially for patients who experience sensitive relapse after initial chemotherapy. In a phase III clinical trial, topotecan significantly prolonged median survival to 25.9 weeks compared to 13.9 weeks with best supportive care (BSC). Topotecan demonstrated a partial response rate of 7% and 44% stable disease, with improved symptom control and slower quality-of-life deterioration. However, hematologic toxicities were common, including grade 4 neutropenia, thrombocytopenia, and anemia [207]. However, the clinical benefit of topotecan remains debated, with some studies suggesting that alternative drugs may achieve similar outcomes. While topotecan has shown effectiveness in certain cases, its efficacy compared to other treatments in sensitive cases is less clear. Additionally, combination therapies have been reported to yield better response rates, which complicates its role as a second-line option [208].

Belotecan has emerged as a promising alternative to topotecan, with studies indicating a favorable toxicity profile. A randomized phase IIb trial compared belotecan and topotecan in 164 patients with sensitive-relapsed SCLC and revealed that the overall response rate (ORR) was higher with belotecan (33%) compared to topotecan (21%, *p* = 0.09). Additionally, median overall survival (OS) was significantly longer with belotecan at 13.2 months versus 8.2 months with topotecan (HR = 0.69, 95% CI: 0.48–0.99). Although progression-free survival (PFS) showed no significant difference between the two groups, belotecan potentially offered a better tolerability profile [209]. As a second-line therapy for refractory-relapsed SCLC, the ORR for belotecan was reported at 14%, with a progression-free survival (PFS) of 1.6 months, suggesting modest benefits [209]. Irinotecan, while primarily used in combination regimens, has also been evaluated as a monotherapy for recurrent SCLC. A systemic analysis highlighted its effectiveness, particularly in patients who had previously responded to irinotecan-containing regimens, with a reported incidence of grade 3/4 toxicities being manageable. The strategy of maintenance chemotherapy with irinotecan has been explored, although its long-term benefits in SCLC remains controversial [210].

More recently, targeting antibody–drug conjugates (ADCs), such as sacituzumab govitecan, has shown promising results in metastatic SCLC [211]. Sacituzumab govitecan is a humanized anti-Trop-2 antibody conjugated by a small-molecule metabolite of irinotecan, SN-38. In a phase II trial, involving 50 heavily pretreated patients, the ADC targeting resulted in tumor shrinkage in 60% of patients, with an overall response rate (ORR) of 14% and a median response duration of 5.7 months, highlighting its potential as a novel therapeutic option [211].

### 7.3. Topoisomerase II Inhibitors

Topoisomerase II inhibitors, such as etoposide, are integral to the first-line treatment of SCLC, typically used in combination with platinum agents (carboplatin or cisplatin). Etoposide induces DNA damage by stabilizing the topoisomerase II-DNA complex, which prevents the re-ligation of DNA strands and ultimately leading to cell death [212]. The drug has been a mainstay in the treatment of both limited-stage and extensive-stage SCLC. The combination of etoposide with platinum-based agents, such as carboplatin or cisplatin, has been established as a standard treatment regimen for both limited-stage and extensive-stage SCLC, yielding response rates exceeding 70% and significantly prolonging median survival times [213]. Despite the initial efficacy of etoposide therapies in SCLC, the development of drug resistance poses a significant challenge. Resistance can arise due to various factors, including the upregulation of the Nrf2 pathway, upregulation of multidrug resistance-associated proteins, and alterations in signaling pathways, particularly those involving MAPK and excision repair cross-complementing 1 (ERCC1) [214,215,216]. Consequently, there is a pressing need to explore alternative therapeutic strategies for patients with relapsed SCLC, particularly those who do not respond to conventional chemotherapy.

Amrubicin, a synthetic anthracycline, is another topoisomerase II inhibitor that is currently approved in Japan. A randomized phase III trial comparing amrubicin/cisplatin (AP) with etoposide/cisplatin (EP) as first-line treatment in SCLC demonstrated that AP was non-inferior to EP, with a median overall survival of 11.8 months for AP and 10.3 months for EP, though the difference was not statistically significant. Progression-free survival was 6.8 months for AP versus 5.7 months for EP [217]. Another key phase III trial compared amrubicin to topotecan as a second-line treatment for SCLC. Although the study revealed that while amrubicin did not significantly improve overall survival (OS) compared to topotecan (7.5 vs. 7.8 months), it did show some benefit in progression-free survival (PFS) (4.1 vs. 3.5 months) and response rate (31.1% vs. 16.9%) [218]. Although these studies have demonstrated the viability of amrubicin in SCLC management, its clinical utility is tempered by its adverse event profiles, with neutropenia being the most common, and there is a lack of significant survival benefits over existing treatments [219].

### 7.4. DLL3 Inhibitors

Delta-like ligand 3 (DLL3) is not typically expressed in normal tissues but is prevalent in SCLC, making it an ideal therapeutic target. Clinical trials of DLL3-targeting therapies, such as antibody–drug conjugates (ADCs) and bispecific T-cell engagers (BiTEs), have shown varying degrees of success. Rovalpituzumab tesirine (Rova-T), an anti-DLL3 ADC, initially showed promise by reducing tumor burden in preclinical and early clinical trials. However, despite these early successes, the drug’s toxic pyrrolobenzodiazepine payload caused significant toxicity, and it ultimately failed to improve overall survival in later-phase trials, leading to its discontinuation [220].

In contrast, tarlatamab, a DLL3-targeting BiTE, has shown more durable efficacy. In the pivotal DeLLphi-301 phase II trial, tarlatamab achieved an objective response rate (ORR) of 40% at a 10 mg dose, with a median progression-free survival (PFS) of 4.9 months, demonstrating sustained activity in patients with relapsed SCLC. Notably, the safety profile was manageable, with cytokine release syndrome (CRS) being the most common adverse event [221]. More recently, in an extended follow-up of a phase I DeLLphi-300 study involving a biweekly 10 mg dose of tarlatamab, the authors achieved a 35.3% objective response rate (ORR), with a median duration of response (DOR) of 14.9 months and an unprecedented median overall survival (OS) of 20.3 months. Its efficacy surpassed both second-line treatments and frontline chemo-immunotherapy for extensive-stage SCLC. The study also demonstrated potent intracranial antitumor activity in patients with brain metastases, highlighting the need for further investigation into tarlatamab’s ability to cross the blood–brain barrier and its underlying mechanism of action [222]. Tarlatamab, which received accelerated FDA approval in May 2024 as a promising treatment for patients with extensive-stage SCLC, offers hope for improved outcomes. However, its continued approval may depend on demonstrating clinical benefit in confirmatory trials, such as the ongoing DeLLphi-304 trial, where the drug is expected to meet its primary endpoints [223].

### 7.5. RNA Polymerase II Inhibitors

Lurbinectedin, an RNA polymerase II inhibitor, has emerged as a promising therapy for small-cell lung cancer (SCLC), particularly in relapsed cases. It is a selective inhibitor of oncogenic transcription and can also affect the tumor microenvironment by targeting tumor-associated macrophages (TAMs) and reducing inflammatory chemokines such as CCL2 [224]. Lurbinectedin received FDA approval on 15 June 2020 for treating adult patients with metastatic SCLC who have progressed following platinum-based chemotherapy, marking the first major advancement in second-line treatment for SCLC in over two decades [225]. The accelerated approval from the FDA was based on encouraging data from a phase II trial, which demonstrated an objective response rate (ORR) of 35.2%, a progression-free survival (PFS) of 3.5 months, and an overall survival (OS) of 9.3 months. Additionally, the treatment was more effective in platinum-sensitive patients (treatment-free interval (TFI) ≥ 90 days), showing a higher overall response rate (ORR) of 45% and a median progression-free survival (PFS) of 4.6 months, compared to an ORR of 22.2% and median PFS of 2.6 months in patients with platinum-resistant disease (TFI < 90 days). Overall survival (OS) was also more favorable in sensitive patients at 11.9 months, compared to 5.0 months in resistant patients, demonstrating better outcomes for sensitive disease [226]. The drug also showed a favorable safety profile, with manageable side effects, including neutropenia and fatigue, which are common in this class of agents.

Lurbinectedin’s success in the clinic has led to its further evaluation in phase III trials as a monotherapy and in combination with other agents such as doxorubicin. However, the ATLANTIS trial, which combined lurbinectedin with doxorubicin, did not show a statistically significant improvement in overall survival compared to standard chemotherapy [227]. Fudio lab conducted an exposure-response analysis to evaluate the individual contributions of lurbinectedin and doxorubicin to the antitumor effects observed in the ATLANTIS trial. Using this model-based, exposure-driven approach, lurbinectedin at 3.2 mg/m^2^ as a single agent showed that the drug outperformed both topotecan and CAV (cyclophosphamide/doxorubicin/vincristine) in the overall population [228]. Currently, lurbinectedin’s accelerated approval is conditional upon confirmatory trials such as the phase III LAGOON trial to verify clinical benefits demonstrating its efficacy.

### 7.6. HDAC Inhibitor

HDAC inhibitors, which target the regulation of gene expression and protein function by acetylating histones and non-histone proteins, have been investigated for their potential to induce tumor cell death and enhance the effects of chemotherapy in SCLC. In non-randomized phase II clinical trials, Panobinostat, a pan-HDAC inhibitor, was tested as a monotherapy in pretreated SCLC patients. The safety profile of Panobinostat was acceptable, with the most common toxicities being mild gastrointestinal disorders and thrombocytopenia. The trial showed some cases of tumor shrinkage (with two of 19 patients showing 30% tumor shrinkage) and stable disease, but the overall efficacy was limited, and the study was prematurely closed due to insufficient activity [229].

The limited success of Panobinostat as a standalone treatment prompted researchers to explore the potential of combining HDAC inhibitors with other therapeutic agents, particularly topoisomerase inhibitors. Preclinical studies by Gray et al. showed that HDAC inhibitors such as vorinostat and mocetinostat induced cell growth inhibition and apoptosis in SCLC cell lines. Synergistic effects were observed when vorinostat was followed by topoisomerase inhibitors like etoposide or topotecan, while antagonist effect was observed on concurrent administration. However, mocetinostat combined with amrubicin or epirubicin demonstrated both concurrent and sequential synergy, leading to enhanced caspase activation compared to single-agent treatments [230]. However, these promising outcomes in preclinical models have not progressed to advanced clinical trials for SCLC.

Despite these setbacks, the development of dual-targeted HDAC inhibitors, such as JBI-802, presents new opportunities for improving treatment outcomes in SCLC. JBI-802 is a small-molecule drug designed to inhibit two epigenetic targets of the CoREST complex: HDAC6 and lysine-specific demethylase 1 (LSD1). The drug demonstrated ability to inhibit neuroendocrine tumor growth in both normal and MYC-amplified variants. Currently, JBI-802 is undergoing phase II clinical trials, having received orphan drug designation for treating small-cell lung cancer (SCLC) and acute myeloid leukemia (AML) [231].

### 7.7. PARP Inhibitors

Numerous PARP inhibitors, including pamiparib (BGB-290), veliparib (ABT-888), Olaparib, and talazoparib (BMN-673), are currently being studied in clinical trials for SCLC [232,233,234,235,236,237], although their benefits appear to be limited and context-dependent. In the phase III trial of niraparib as maintenance therapy after first-line chemotherapy in extensive-stage SCLC, the drug demonstrated a modest improvement in progression-free survival (PFS) compared to placebo (1.54 vs. 1.36 months). However, the trial did not meet its primary overall survival (OS) endpoint, with no significant difference in OS between the niraparib and placebo groups (9.92 vs. 11.43 months). While the drug was well tolerated, common adverse effects included hematologic toxicities such as anemia and neutropenia [238].

Veliparib has been tested in combination with standard chemotherapy, showing some potential in improving outcomes in SCLC. In a phase I trial, veliparib combined with carboplatin and etoposide demonstrated an overall response rate (ORR) of 64% in extensive-stage SCLC patients, with dose-limiting toxicities primarily related to hematological adverse effects, including neutropenia and thrombocytopenia [239]. In a subsequent phase II trial, while the addition of veliparib resulted in modest improvement in PFS of 5.8 months versus 5.6 months for the control group, the difference in overall survival was not significant (10.1 months for veliparib vs. 12.4 months for the control). These findings suggest that veliparib’s efficacy may be limited, particularly in unselected patient populations [240].

Olaparib, another PARP inhibitor, has been investigated as a monotherapy in the phase II STOMP trial, which evaluated it as a maintenance treatment for SCLC patients who had responded to chemotherapy. In this trial, Olaparib did not provide a statistically significant improvement in PFS compared to placebo (3.7 vs. 2.5 months), and overall survival outcomes were also not significantly improved [241]. Similarly, in a more recent phase II biomarker-driven umbrella study, Olaparib alone and in combination with the ATR inhibitor ceralasertib demonstrated limited efficacy, with an ORR of just 6.7% for Olaparib monotherapy and 3.8% for the combination. The disease control rate was higher for the combination therapy arm (42.3%) compared to monotherapy (33.3%), suggesting its potential in combination treatments. These results highlight the need for evaluating Olaparib in multiple combinations while improving patient selection strategies [242].

Despite these modest results, preclinical studies have shown that combining PARP inhibitors with other agents, such as immune checkpoint inhibitors or DNA damage response inhibitors like WEE1 or ATR inhibitors, may enhance efficacy [243,244]. For example, Lallo et al. demonstrated that combining Olaparib with WEE1 kinase inhibitors (AZD1775) can significantly improve outcomes in SCLC models, suggesting that such combinations may be more effective than monotherapy [243].

### 7.8. CHK1 Inhibitors

CHK1 inhibitors have gained attention as a potential treatment for small-cell lung cancer (SCLC) due to their ability to disrupt the DNA damage response (DDR) and enhance tumor sensitivity to chemotherapy. Among these inhibitors, prexasertib has undergone the most extensive clinical evaluation in SCLC. Despite initial promise in early trials, a phase II study evaluating prexasertib as monotherapy in platinum-sensitive and platinum-refractory patients with extensive-stage SCLC (ED-SCLC) yielded disappointing results. In platinum-sensitive patients, the objective response rate (ORR) was only 5.2%, with a disease control rate (DCR) of 31%. For platinum-refractory patients, there were no objective responses, and the DCR was 20%. Both groups showed a median progression-free survival (PFS) of 1.4 months, with overall survival (OS) ranging from 3.15 to 5.42 months. Given these modest outcomes, the development of prexasertib as a monotherapy was not pursued further [245]. However, recent preclinical studies demonstrated that combining prexasertib with lurbinectedin, led to enhanced tumor cell death and increased DNA damage, indicating a synergistic effect that could improve outcomes [246].

Additionally, research by Zhao et al. revealed that resistance to CHK1 inhibitors may arise through the upregulation of Wee1, a kinase involved in cell cycle regulation. Combining CHK1 inhibitors with Wee1 inhibitors could potentially overcome this resistance, offering a more robust and effective therapeutic strategy for SCLC [247]. Furthermore, Sen et al. demonstrated that MYC overexpression in SCLC models is linked to increased sensitivity to prexasertib, particularly when used in combination with cisplatin or the PARP inhibitor Olaparib, highlighting the importance of selecting patients based on genetic biomarkers to predict therapy responsiveness in specific patient populations [123]. SRA-737, an oral small-molecule CHK1 inhibitor, is undergoing clinical trials for various solid tumors, including small-cell lung cancer (SCLC) (NCT0279964, NCT02797977) [248].

### 7.9. Aurora Kinase Inhibitors

The role of Aurora kinases in mitosis is critical, as their overexpression is often associated with poor prognosis in various cancers, including SCLC, where inactivation of the RB1 tumor suppressor gene is prevalent. This inactivation increases the susceptibility of cancer cells to mitotic disruption, making them prime candidates for targeted therapies [128]. Specifically, Barasertib, a selective Aurora kinase B inhibitor, has shown promise in inducing apoptosis in multiple SCLC cell lines, and inhibit tumor growth in mouse xenograft models with cMYC amplification [131]. However, clinical trials with the AURKB inhibitor AZD2811 have shown limited efficacy. For instance, in one trial, AZD2811NP (an encapsulated slow-release formulation of AZD2811) led to a median progression-free survival (PFS) of only 1.6 months, with no objective responses. In addition, suspected serious adverse effects, including neutropenia, particularly when combined with immune checkpoint inhibitors like durvalumab, led to the early termination of trials such as SUKSES-N5 [249]. Alisertib, an AURK A inhibitor, has been more extensively studied in clinical trials for SCLC and has received orphan drug designation from the FDA for the treatment of extensive-stage small-cell lung cancer (ES-SCLC). Preclinical models indicated higher sensitivity in tumors with high c-Myc expression or loss of RB1.

Phase I/II clinical trials of alisertib as monotherapy or in combination with paclitaxel for relapsed/refractory solid tumors, including SCLC, showed response rates of 21–22%, with common-grade ≥3 adverse events being neutropenia, febrile neutropenia, and leukopenia [250]. In clinics, high c-Myc expression or mutation in RB1, RBL1, RBL2, or CDK6 demonstrated strong correlation with improved progression-free survival (PFS) and overall survival (OS) in the alisertib treatment arm [251]. Further studies have investigated the combination of Alisertib with the third-generation Epidermal Growth Factor Receptor (EGFR) tyrosine kinase inhibitor osimertinib in EGFR-mutated lung cancer. This combination achieved an overall response rate of 9.5% and a median progression-free survival (PFS) of 5.5 months.

However, the disease control rate (DCR) was notably higher at 81%, indicating that the combination therapy may offer improved disease stabilization [252]. In another study, a randomized phase II trial of paclitaxel combined with the selective Aurora kinase A inhibitor, Alisertib (MLN8237; Takeda) demonstrated significant efficacy in comparison to paclitaxel plus placebo as a second-line treatment for small-cell lung cancer (SCLC) [251].

### 7.10. BCL-2 Inhibitors

BCL-2 inhibitors, particularly venetoclax and navitoclax, have been investigated in small-cell lung cancer (SCLC) due to the frequent overexpression of BCL-2, a key regulator of apoptosis. Venetoclax, a selective BCL-2 inhibitor, has shown significant promise, particularly in SCLC patients with high BCL-2 expression. Preclinical studies showed that venetoclax induces apoptosis binding to BCL-2, displacing the pro-apoptotic protein BIM from the BCL-2 complex. This disruption allows BIM to activate the apoptotic pathway, leading to cell death and reduction in tumor growth in BCL-2-expressing SCLC models. Thus, high BCL-2 expression is predictive biomarker for venetoclax sensitivity, making it a potential targeted therapy for certain SCLC patients [253].

Navitoclax, a dual inhibitor of both BCL-2 and BCL-xL, has also been studied in SCLC. In a phase II trial involving 39 patients with relapsed SCLC, navitoclax demonstrated limited clinical efficacy. Only one patient (2.6%) achieved a partial response, while nine patients (23%) experienced stable disease. The median progression-free survival (PFS) was 1.5 months, and overall survival (OS) was 3.2 months. Additionally, navitoclax was associated with significant grade III–IV thrombocytopenia in 41% of patients, which posed challenges in optimizing the dosing due to its on-target toxicity in platelets [254]. Given these results, navitoclax’s use as a single agent has been limited, prompting the exploration of combination strategies to improve its effectiveness. For example, combining navitoclax with the histone deacetylase (HDAC) inhibitor vorinostat has shown potential in overcoming resistance in SCLC cell lines by promoting apoptosis through the modulation of key apoptotic proteins, like Noxa and BIM, even in cells that are resistant to navitoclax alone [255].

To address the platelet toxicity challenges associated with navitoclax, a novel approach using proteolysis-targeting chimeras (PROTACs) has been developed. A dual degrader, 753b, was designed to selectively degrade both BCL-2 and BCL-xL. Preclinical studies demonstrated that 753b significantly reduced tumor growth in SCLC models while avoiding the thrombocytopenia observed with navitoclax, as it spares platelets. This approach showed superior efficacy compared to navitoclax and required lower doses to achieve similar tumor regression, making it a promising option for safer and more effective treatment [256].

**Table 1:** Representative clinical trials against Small-cell lung cancer (SCLC).

	PD-L1 inhibitors			
Drug/Drug Combinations	Phase Trial	Results	Trial IDs	References
Pembrolizumab in combination with etoposide and platinum-based chemotherapy	KEYNOTE-604 trial, a pivotal phase III study	1. Patients who received pembrolizumab significantly improved their overall survival (OS) and progression-free survival (PFS) compared to those who received a placebo. 2. The study reported a median OS of 12.9 months for the pembrolizumab group, compared to 10.5 months for the placebo group.	NCT03066778	[200]
Combination of atezolizumab with carboplatin and etoposide	IMpower133 trial, randomized, double-blind, phase I/III	1. The atezolizumab group’s median OS was 12.3 months, while the control group was 10.3 months, underscoring the effectiveness of atezolizumab in treating extensive-stage SCLC (ES-SCLC). 2. The safety profile was also favorable, with manageable adverse events.	NCT02763579	[201]
Durvalumab in combination platinum–etoposide	CASPIAN phase III trial	1. Median OS of 13.0 months for the durvalumab group in comparison to 10.3 months for the chemotherapy-only group, demonstrating enhanced survival rates in ES-SCLC patients.	NCT03043872	[202]
	**Topoisomerase I Inhibitors**			
Topotecan	Phase III clinical trials	1. The median survival increased to 25.9 weeks from 13.9 weeks with optimal supportive care (BSC). 2. Topotecan demonstrated a partial response rate of 7% and 44% stable disease, together with improved symptom control and slower quality-of-life deterioration. 3. Also demonstrated hematologic toxic side effects such as grade 4 neutropenia, thrombocytopenia, and anemia etc.	NCT00276276	[207]
Comparison between belotecan and topotecan	Randomized phase IIb trial	1. A trial with 164 patients with sensitive-relapsed SCLC showed that belotecan had a higher overall response rate (ORR) (33%) than topotecan (21%, *p* = 0.09). 2. Median overall survival (OS) was significantly longer with belotecan at 13.2 months versus 8.2 months with topotecan along with a better tolerability profile.	NCT01497873	[209]
Sacituzumab govitecan, humanized anti-Trop-2 antibody conjugated by a small-molecule metabolite of irinotecan, SN-38	Phase II trial	1. The trial involved 50 heavily pretreated SCLC patients and demonstrated significant tumor shrinkage in 60% of them, with an overall response rate (ORR) of 14% and a median response duration of 5.7 months.	NCT01631552	[211]
	**Topoisomerase II Inhibitors**			
Comparison between amrubicin/cisplatin (AP) with etoposide/cisplatin (EP)	Randomized phase III trial	1. AP was non-inferior to EP, with a median overall survival of 11.8 months for AP and 10.3 months for EP, though the difference was not statistically significant. 2. Progression-free survival was 6.8 months for AP versus 5.7 months for EP.	NCT00660504	[217]
Comparison of amrubicin to topotecan	Phase III trial	1. Amrubicin did not significantly improve overall survival (OS) compared to topotecan (7.5 vs. 7.8 months), but it did show some benefit in progression-free survival (PFS) (4.1 vs. 3.5 months) and response rate (31.1% vs. 16.9%).	NCT00547651	[218].
	**DLL3 Inhibitors**			
Tarlatamab	DeLLphi-301 Phase II trial	The treatment demonstrated sustained activity in patients with relapsed SCLC, achieving an objective response rate (ORR) of 40% at a 10 mg dose and a median progression-free survival (PFS) of 4.9 months. 2. The common adverse symptom is the cytokine release syndrome (CRS), which is the most common adverse event.	NCT05060016	[221]
Tarlatamab	Extended follow-up of Phase I DeLLphi-300	1. A biweekly 10 mg dose of tarlatamab achieved a 35.3% objective response rate (ORR), with a median duration of response (DOR) of 14.9 months and an unprecedented median overall survival (OS) of 20.3 months. 2. Its efficacy surpassed both second-line treatments and frontline chemoimmunotherapy for extensive-stage SCLC.	NCT03319940	[222]
	**RNA Polymerase II Inhibitors**			
Lurbinectedin	Phase II trial	1. The treatment demonstrated an objective response rate (ORR) of 35.2%, a progression-free survival (PFS) of 3.5 months, and an overall survival (OS) of 9.3 months. Additionally, the treatment was more effective in patients with platinum-sensitive patients (treatment-free interval (TFI) ≥ 90 days), showing a higher overall response rate (ORR) of 45% and a median progression-free survival (PFS) of 4.6 months, compared to an ORR of 22.2% and median PFS of 2.6 months in patients with platinum-resistant disease (TFI < 90 days). 2. Overall survival (OS) was also more favorable in sensitive patients at 11.9 months, compared to 5.0 months in resistant patients, demonstrating better outcomes for sensitive disease with minimal side effects.	NCT02454972	[226]
	**HDAC inhibitor**			
Panobinostat	Non-randomized Phase II clinical trials	The trial was conducted on pretreated SCLC patients. The results showed some cases of tumor shrinkage. (with two of 19 patients showing 30% tumor shrinkage) and stable disease, but the overall efficacy was limited, and the study was prematurely closed due to insufficient activity.	NCT01222936	[229]
	**PARP inhibitors**			
Niraparib	Phase III trial	1. In extensive-stage SCLC, the drug demonstrated a modest improvement in progression-free survival (PFS) compared to placebo (1.54 vs. 1.36 months). However, the trial did not meet its primary overall survival (OS) endpoint, with no significant difference in OS between the niraparib and placebo groups (9.92 vs. 11.43 months). 2. Despite the drug’s good tolerance, common side effects included hematologic toxicities like anemia and neutropenia.	ZL-2306-005	[238]
Veliparib	Phase I trial	1. Combining veliparib with carboplatin and etoposide showed an overall response rate (ORR) of 64% in patients with advanced SCLC. 2. The most common toxicities were related to the blood, specifically neutropenia and thrombocytopenia.	NCT02289690	[239]
Combination of Olaparib and ATR inhibitor ceralasertib	Phase II biomarker-driven umbrella study	The disease control rate was higher for the combined therapy arm (42.3%) compared to monotherapy (33.3%), suggesting its potential in combination treatments.	NCT03009682	[242]
Alisertib as monotherapy or in combination with paclitaxel	Phase I/II clinical trials	The trial showed response rates of 21–22%, with common grade ≥3 adverse events being neutropenia, febrile neutropenia, and leukopenia.	NCT06095505	[250]
	**BCL-2 Inhibitors**			
Navitoclax	Phase II study	Thirty-nine patients with relapsed SCLC participated in the trial, with only one (2.6%) achieving a partial response and nine (23%) experiencing stable disease. The median progression-free survival (PFS) was 1.5 months, and overall survival (OS) was 3.2 months.	NCT00445198	[254]

## 8. Discussion

The current treatment landscape for small-cell lung cancer (SCLC) has made significant progress, but crucial gaps still hinder improved patient outcomes. Despite recent advances, such as incorporating immune checkpoint inhibitors (ICIs) like atezolizumab and durvalumab into first-line therapy for extensive-stage SCLC, overall survival benefits remain modest. Most patients experience rapid relapse after initial chemotherapy, and resistance to both chemotherapy and immunotherapy continues to be a persistent challenge. In one report, researchers aimed to assess the effectiveness of Imatinib mesylate in the treatment of SCLC. The serum-dependent proliferation of SCLC cells has been demonstrated to be inhibited, with an IC_50_ of around 1–5 µmol/L. The inhibitory effect of Imatinib seems to be associated with the presence of KIT expression [257,258]. Nonetheless, this small phase II trial involving 19 SCLC patients treated with Imatinib did not reveal any evidence of antitumor activity. The occurrence of KIT positivity in tumor samples was significantly lower than expected, with rates of 21% compared to the anticipated 70% [259,260]. Additional research is required in the future to evaluate the function of KIT expression in SCLC.

One major gap is the limited availability of therapeutic options beyond the first-line setting, particularly due to the lack of predictive biomarkers that could help tailor immunotherapy more effectively. This is further complicated by the heterogeneity of SCLC, as recent molecular profiling has revealed four distinct transcriptional subtypes (SCLC-A, SCLC-N, SCLC-P, and SCLC-Y), each with unique biological features and therapeutic vulnerabilities. Moreover, these subtypes are highly plastic and exhibit significant intratumoral heterogeneity, complicating the development of targeted therapies. Additionally, systemic toxicity from conventional chemotherapy and immunotherapy remains a critical challenge, often limiting their use and effectiveness. Platinum-based chemotherapy, while essential, causes widespread toxic effects that significantly impact quality of life. To address this, novel targeted drug delivery systems such as antibody–drug conjugates (ADCs) and nanoparticle-based carriers are being developed. These systems aim to deliver drugs directly to tumor cells, minimizing damage to healthy tissues and reducing systemic toxicity. Similarly, nanoparticle-based platforms are being explored to enhance the precision of chemotherapeutic delivery, reducing off-target effects and improving safety.

Looking forward, opportunities for research include exploring novel therapeutic targets, such as DNA damage repair mechanisms, cell cycle checkpoints, and epigenetic modifiers. Biomarker-driven strategies, including liquid biopsies and single-cell analyses, could better capture tumor dynamics and refine precision medicine approaches. Emerging therapies like bispecific antibodies, adoptive cell transfer (ACT), and chimeric antigen receptor (CAR) T-cell therapy offer promising avenues to overcome resistance and improve outcomes in relapsed SCLC. Addressing these gaps—through biomarker discovery, the development of novel combination regimens, refinement of targeted delivery systems, and personalized treatment strategies—will be essential in advancing the management of SCLC.

## 9. Conclusions

SCLC is a highly recalcitrant cancer distinguished by unique molecular patterns along with substantial tumoral heterogeneity [10]. Further investigation is required to pinpoint more effective genes and signaling pathways crucial for the survival and proliferation of SCLC cells. Recent advances in small-cell lung cancer (SCLC) treatment highlight a transformative era in combating this aggressive disease. Breakthrough therapies, including checkpoint inhibitors like pembrolizumab and atezolizumab, and targeted agents such as lurbinectedin and sacituzumab govitecan, have improved survival and addressed treatment resistance. Precision medicine, incorporating biomarkers and personalized approaches, is becoming central to optimizing efficacy while minimizing adverse effects. Emerging treatments targeting novel pathways, like DLL3 and HDAC, show promise, while collaboration among researchers and industry leaders is crucial for translating innovations into clinical practice. Ongoing research into resistance mechanisms and combination therapies offers hope for achieving durable responses and advancing the standard of care.

## Data Availability

No new data were created or analyzed in this study. Data sharing is not applicable to this article.
